# A Comprehensive Review on Valorization of Agro-Food Industrial Residues by Solid-State Fermentation

**DOI:** 10.3390/foods10050927

**Published:** 2021-04-23

**Authors:** Gordana Šelo, Mirela Planinić, Marina Tišma, Srećko Tomas, Daliborka Koceva Komlenić, Ana Bucić-Kojić

**Affiliations:** Faculty of Food Technology Osijek, Josip Juraj Strossmayer University of Osijek, Franje Kuhača 18, HR-31000 Osijek, Croatia; gselo@ptfos.hr (G.Š.); mplanini@ptfos.hr (M.P.); mtisma@ptfos.hr (M.T.); stomas@ptfos.hr (S.T.); dkoceva@ptfos.hr (D.K.K.)

**Keywords:** lignocellulosic biomass, value-added products, bioactive compounds, biofuels, feed

## Abstract

Agro-food industrial residues (AFIRs) are generated in large quantities all over the world. The vast majority of these wastes are lignocellulosic wastes that are a source of value-added products. Technologies such as solid-state fermentation (SSF) for bioconversion of lignocellulosic waste, based on the production of a wide range of bioproducts, offer both economic and environmental benefits. The versatility of application and interest in applying the principles of the circular bioeconomy make SSF one of the valorization strategies for AFIRs that can have a significant impact on the environment of the wider community. Important criteria for SSF are the selection of the appropriate and compatible substrate and microorganism, as well as the selection of the optimal process parameters for the growth of the microorganism and the production of the desired metabolites. This review provides an overview of the management of AFIRs by SSF: the current application, classification, and chemical composition of AFIRs; the catalytic function and potential application of enzymes produced by various microorganisms during SSF cultivation on AFIRs; the production of phenolic compounds by SSF; and a brief insight into the role of SSF treatment of AFIRs for feed improvement and biofuel production.

## 1. Introduction

The term “residue” includes materials that are not intentionally generated in the production process but are not necessarily considered waste [1]. Significant amounts of residues are generated during the processing of plant raw materials that are transformed into final products, e.g., in wine production and grain processing, residues account for about 30% of the processed raw material mass [2,3]. They are treated as waste and their unregulated disposal into the environment can cause serious environmental problems [4]. AFIRs are a wide variety of biomass, including pomace, fruit, and vegetable peels; husks, bran, and germ of cereals; pods; stalks; and pomace left over after oil production. Due to their chemical composition, they are rich sources of high value components such as polysaccharides, proteins (including enzymes), dietary fibers, fatty acids, flavors and aromas, and bioactive compounds [5,6,7,8]. High-value components refer to components that have health-promoting properties and a wide range of potential industrial applications (pharmaceutical, food, and cosmetic industries) due to their biological activity or nutritional value [5,6].

AFIRs are mostly lignocellulosic materials composed of three polymers: cellulose (40–50%), hemicellulose (20–30%), and lignin (20–35%). Lignin is the main component of the cell wall [9,10]. According to numerous studies, SSF is one of the most suitable techniques to obtain the desired biomolecules from lignocellulosic materials. SSF has been extensively studied for potential applications in fuel, food, and feed, as well as in the chemical and pharmaceutical industries [11,12].

By-products from lignocellulosic biomass are an important alternative energy source, playing an important role in the circular bioeconomy. The management of this resource promotes the reuse of raw materials, high industrial production yields, and the generation of minimal waste [13]. The efficient use of natural resources, the development of new technologies, and the improvement of existing ones increase the value of agricultural waste. Their use in the production of biogas, biofuels, biofertilizers, bioactive compounds, and pharmaceuticals is consistent with sustainable development and the business model of using agricultural waste in the bioeconomy. SSF can be applied as a technique of biological processing of different lignocellulosic materials to obtain different products following the concept of the 3-R approach “reduce, reuse, recycle” [14], thus contributing to the circular bioeconomy [15].

This paper provides a comprehensive overview of the possibilities of valorization of lignocellulosic biomass produced in the agro-food industry. Particular attention is paid to the composition of the different types of AFIRs, taking into account that they come in different forms and in many processing routes. SSF experiments on the valorization of AFIRs for the production of enzymes (ligninolytic and hydrolytic enzymes) and a wide number of value-added products through the use of different microorganisms are discussed. In addition, the valorization of lignocellulosic biomass treated with microorganisms for use in feed and biofuel production is presented.

## 2. Agro-Food Industrial Residues

**Figure 1:**
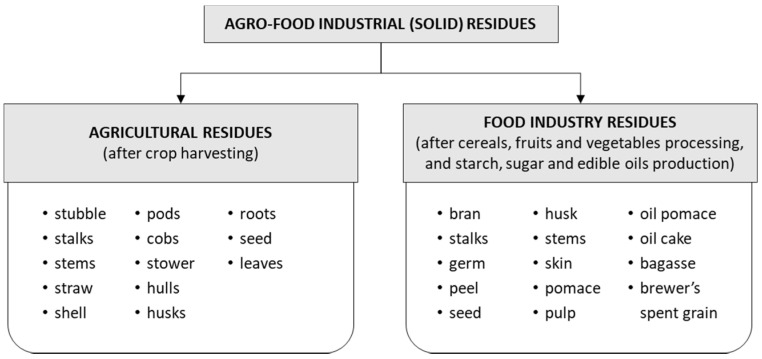
Classification of AFIR.

The increasing expansion of agro-industrial activities in recent decades has resulted in the accumulation of a large amount of lignocellulosic residues (wastes or by-products) worldwide, which are not properly disposed of and thus contribute to climate change, as well as soil, water, and air pollution [16,17]. An estimated one-third (≈1.3 billion tonnes) of food produced for human consumption is wasted annually worldwide [18]. Although some of these residues are used as animal feed, large quantities are disposed of in landfills or incinerated [17]. In general, AFIRs can be divided into agricultural residues and food industry residues (Figure 1). This review does not cover food waste, which includes unsold food, leftovers, and uneaten food from households and restaurants, as well as from large-scale producers such as caterers and supermarkets [19].

### 2.1. Agricultural Residues

Agricultural residues include the waste left on fields after harvesting and the waste after processing of raw materials (pods, stubble, stems, stalks, leaves, shell, straw, seeds, husks, roots, etc.) (Figure 1). Harvest residues represent an alternative to generate large amounts of energy in the future [20]. Some examples of widely used agricultural residues are straw from cereals (wheat, barley, oats, rye, rice, spelt), barley hulls, soybean stalks, corn cobs, corn stalks, sunflower stalks, sunflower seed hulls, etc., and their chemical compositions are presented in Table 1. The type of cereal grains and their residues produced worldwide depends on economic, cultural, and environmental factors. The average annual production of cereals is 2.435 million tonnes (data for the period 2008–2017) [21]. Water availability and temperature are the most important environmental factors that can affect grain production.

### 2.2. Food industry Residues

Residues resulting from the processing of raw materials in the various food industries (fruit industry, beer industry, oil industry, cereal-processing industry) consist of pips, skins, stalks, pomace, oil cake, oil pomace, brewer’s grains, bran, germ, etc. (Figure 1). Grape pomace, apple pomace, wheat bran, rye bran, rice bran, hull-less pumpkin oil cake, hemp oil cake, and flax oil cake are some examples of residues from the food industry that are produced in significant quantities and can be used for various energy sources and to produce a variety of valuable compounds. The chemical composition of common food industry residues is presented in Table 2. EU processed food producers have to comply with the conditions of the EU environmental policy regarding the recycling or disposal of the generated residues [28]. Grape pomace, which is a by-product of wine production, and cereal residues, which are a by-product of grain processing, are representative examples of the production of value-added compounds from industrial food residues [3,33].

*Vitis vinifera* is one of the most widely grown crops in the world, with an average annual production of 71 million tons (data for period 2008–2017) [21]. It is estimated that about 80% of the annual grape yield is processed into wine, while 20–30% of the processed grapes remain as grape pomace (grape marc). It has been found that only 30% of the phenolic compounds are extracted into the wine during winemaking, while 70% of the bioactive phenolic compounds remain in the grape pomace [34]. The reason for this is the high content of polymeric proanthocyanidins in the grape pomace and of bound phenolic compounds in complexes with the proteins, fibers, and polysaccharides, which are difficult to extract unless pretreatment is carried out (acid, alkali, biological treatment, etc.) [35].

Grape pomace consists of seeds, skin, and sometimes stems. According to the literature, the ratio of grape seeds in grape pomaces varies in the range of 15–52%_db_, while the proportion of grape skins can be as high as 65%_db_ [2]. Grape pomace can be a good substrate in SSF due to its composition, changing the properties of grape pomace. According to the available studies, grape pomace is generally considered a good source of polyphenolic compounds [36], although there are studies on the production of enzymes [33] and biofuels from grape pomace.

The term cereals refers to nine species: wheat, rye, barley, oats, rice, millet, corn, sorghum, and triticale (a hybrid of wheat and rye). Cereals consists of hull and kernel, where kernel consists of bran, germ, and endosperm [31]. The cereal processing industry produces 30% of the residues (hull, bran), which are mainly represented by bran. The bran is separated during milling of the grain [32]. Wheat and oat whole grains are known to be a rich source of phenolic compounds that exhibit significant antioxidant activity. The predominant phenolic compounds are phenolic acids and flavonoids, with the highest concentrations found in the bran, but still they have reduced bioavailability [3].

### 2.3. Chemical Composition of AFIRs

On the basis of the carbon source, Mitchell et al. [53] classify substrates for SSF into three groups: starch substrates (contain starch as the main carbon source), lignocellulosic substrates (contain cellulose and lignocellulose as the main carbon source), and substrates containing mainly soluble sugars. Most AFIRs are classified as lignocellulosic biomass. Generally, AFIRs contain a high content of polysaccharides such as cellulose, hemicellulose, and lignin, but also contain other nutrients such as proteins, lipids, pectin, and polyphenols [54]. A literature overview of the chemical composition of AFIRs derived from various sources is presented in Table 1 and Table 2.

Such material is complex and heterogeneous, as can be seen from its chemical composition. Agronomic measures of cultivation, weather conditions, variety, harvesting methods, storage conditions, and the analytical methods used to measure the individual components are all factors that influence the chemical composition of the material.

Commonly used analytical methods to determine the chemical composition of such material are ash determination (total combustion in a muffle furnace), determination of total organic carbon [55], total proteins (Kjeldahl method) [56], free fats (direct solvent extraction), neutral detergent fibers (NDF), acid detergent fibers (ADF), acid detergent lignin (ADL) determination by Van Soest [55], acid-insoluble lignin (Klason lignin) determination by gravimetric assay, total polyphenolic compounds determination by the Folin–Ciocalteu colorimetric method [57], and individual polyphenolic compounds determination by UHPLC [58], among others.

Lignocellulosic structure of AFIRs can be disrupted by biocatalytic activities of different microorganisms. In order for one to choose the best method (microorganism, type of cultivation, process conditions, etc.) for its reuse, such residues should be firstly chemically characterized.

## 3. Solid-State Fermentation (SSF)

### 3.1. General

SSF is a fermentation process in which microorganisms grow on moist, solid material under controlled conditions, without the presence of free water or with a minimal amount of free water. Inert or non-inert materials can be used as solid substrate in SSF processes [59]. AFIRs are among the non-inert solid substrates that serve as nutrients for microbial growth and metabolite production. After fermentation, they can also be the product of fermentation, used for feed or biofuel production.

The microorganisms used in SSF are filamentous fungi, yeasts, and bacteria. Due to their physiological, biochemical, and enzymatic properties, filamentous fungi (multicellular organisms) are the most commonly used, especially those from fungal kingdom sub-division *Basidiomycota* and *Ascomycota* [60,61]. In order to develop a reliable and repeatable SSF process, researchers should carry out this process in specific types of bioreactors under controlled conditions, such as tray bioreactors, rotating disc reactors, fixed bed bioreactors, column bioreactors, air pressure pulsation solid state bioreactors, rotating horizontal drum bioreactors, stirred drum bioreactors, fluidized bed bioreactors, air-lift bioreactors, and immersion bioreactors [8,62]. The most suitable type of bioreactor for SSF scale-up is the tray bioreactor. It is a traditional type of bioreactor used in SSF, most commonly in laboratory research for enzyme production [63], for lignin degradation [62,63] for the application of biologically pretreated material in the process of biogas production [55]. It is also used in commercial processes in various industries, such as the production of fermented foods such as tempeh [8] and the production of various enzymes [64]. This is due to its simple design and ease of use [65].

The advantages of SSF over submerged fermentation (SmF) are its similarity to the natural habitat of microorganisms, higher productivity, lower cost (due to the use of cheap agro-industrial residues as substrates), lower water consumption, lower use of chemicals, lower generation of waste streams, and lower energy consumption [60,66,67,68]. Despite these advantages, there are some important technological concerns that need to be considered in order to improve the overall SSF process. Some of them are problems caused by the heterogeneity of the system, such as heat and mass transfer resistance; separation of the microorganisms from the substrate; and sampling problems during fermentation for continuous monitoring of the chemical composition of the substrate and/or product accumulation [69].

The most important factors that have effect on the efficiency of SSF process are substrate (chemical composition, humidity, and particle size), inoculum (concentration, age, and morphology), external carbon and/or nitrogen addition, addition of a specific enzyme’s inducers for microorganism’s growth and/or desired metabolite production, mixing, temperature, pH, and oxygen concentration.

The basic information of substrates and microorganisms that are commonly used in SSF are provided further in this paper.

### 3.2. Substrates Used in SSF

The choice of substrate is usually determined by its cost and availability, its chemical composition (Table 1 and Table 2), and its suitability to be converted into a particular product via biochemical pathways. Depending on the objective (production of the desired enzyme, production of the desired phenolic compounds, organic acids or other valuable product, use for biofuel production, use for feed processing), it is important to know the chemical composition of the substrate and to select a suitable microorganism. If the substrate does not contain the required amounts of nutrients, some macro- and micronutrients are added for optimal growth of the microorganisms [70,71]. Macronutrients (carbon, nitrogen, oxygen, hydrogen, sulfur, phosphorus, Mg^2+^, and K^+^) are needed in concentrations greater than 10^−4^ M, whereas carbon in the growth medium is the main source of energy. Microelements (Mo^2+^, Zn^2+^, Cu^2+^, Mn^2+^, Ca^2+^, Na^+^) and vitamins, growth hormones, and metabolic precursors are needed in concentration less than 10^−4^ M [60]. AFIRs are a source of carbon, nitrogen, and nutrients and can therefore serve as solid carriers suitable for nutrient absorption and biomass growth [71]. Sometimes it is necessary to combine several different residues according to their chemical composition and to use such a mixture as a substrate to ensure sufficient nutrients for the optimal growth of microorganisms [11]. For SSF, the moisture content of the substrate is one of the most important operating parameters that affects the whole fermentation process. If the moisture content is too high, the interstitial spaces of the solid material will be filled with water and gas diffusion will be restricted. On the other hand, if the moisture content is too low, the growth of microorganisms will be impaired. The optimum moisture content depends on the substrate and the microorganism and changes during the fermentation process [72]. The final water content is the sum of the initial water content and the water produced by the metabolism of the microorganisms minus the water removed by evaporation. Water partially evaporates, and at the same time it is produced by the metabolism of the microorganisms [73]. If the production of metabolic water is greater than the evaporated water, then the water content is reduced [62].

Particle size and shape of the substrate can affect the accessibility of nutrients to the microorganism. Smaller particle size causes the smaller inter-particle spaces and the greater pressure drops when air flows through the substrate mass. The particles with a larger surface area tend to be contiguous with the flat surfaces and thus actually exclude oxygen, limiting the growth of microorganisms [74]. The particle size range of the substrate used in SSF is usually between 0.25 and 7.5 mm, depending on the type of substrate used [75,76,77,78,79].

### 3.3. Microorganisms Used in SSF

Filamentous fungi, yeasts, and bacteria can be used in the SSF process to produce value-added compounds. Unicellular organisms such as bacteria and yeast grow as a biofilm, while multicellular filamentous organisms grow in the form of a mycelium, which is comprised of aerial and penetrative hyphae. If the layer of hyphae is thick, then water moves by capillary action from the substrate, resulting with layer into a moist biofilm. A biofilm can also be formed in the case when the bed is mixed, since mixing causes squashing of aerial hyphae onto the surface of the substrate [8]. The most commonly used microorganisms in SSF are filamentous fungi. The choice of microorganism depends on the desired end product, while the choice of substrate is an important parameter for the successful growth of the selected microorganism [80]. The microorganisms can be used as single cultures, as identifiable mixed cultures, or as a consortium of mixed indigenous microorganisms. Many factors can affect the growth of microorganisms, such as the moisture content and properties of the substrate (chemical composition, particle size, height of the substrate layer), temperature, aeration, mixing, initial concentration, and age of the microorganisms [80,81]. Microbial growth usually results in the release of metabolic heat. High temperatures can lead to denaturation of enzymes and affect metabolite production. Since SSF occurs in the absence of free water, it is difficult to dissipate the heat generated during microbial growth due to the limited thermal conductivity of the solid substrate and the low heat capacity of the air. The difficulties in controlling the temperature in SSF can become even more pronounced when the process is carried out on a large scale.

#### 3.3.1. Filamentous Fungi

Filamentous fungi comprise almost the entire kingdom of fungi and represent the most diverse group of microorganisms that produce filamentous hyphae. SSFs mimic the natural habitats of filamentous fungi and grow on the surface of and within substrate parts due to their hyphal growth. The term filamentous fungi is used in contrast to yeasts, which are essentially unicellular fungi [8]. Filamentous fungi have great potential for the production of enzymes with high commercial value and/or many different valuable compounds by SSF as a result of enzymatic activity (Table 3) [5,11,80].

There are numerous studies on the cultivation of filamentous fungi of the genus *Trametes* under SSF conditions on different AFIRs for the purpose of laccase production. For example, when comparing the cultivation of *T. hirsuta* on grape seeds in two types of laboratory-scale bioreactors (immersion and tray bioreactors), one study found the tray bioreactor to be better in terms of the highest laccase activity achieved (2.5 × 10^5^–3 × 10^5^ nkat/L) [59]. Studies by Bucić-Kojić et al. [82] have shown that *T. versicolor*, in addition to laccase production, is also effective in the recovery of phenolic acids during SSF on corn silage in laboratory jars. Thanks to the complex enzyme system of *T. versicolor*, which successfully degrades lignin, there was an increase in the extraction yield of phenolic acids (syringic acid, vanillic acid, *p*-hydroxybenzoic acid, caffeic acid) during the biological treatment compared to the initial biologically untreated corn silage.

Filamentous fungi of the genera *Aspergillus* and *Rhizopus* are commonly used in studies investigating the SSF process because they produce several enzymes with broad substrate specificity that are stable at lower pH and high temperatures and play an important role in the hydrolysis of phenolic conjugates [83]. Dulf et al. [83] investigated the cultivation of *A. niger* and *R. oligosporus* on apricot pomaces under SSF conditions. The obtained results showed that there is an increase in total phenolic content when *R. oligosporus* was used until the ninth day of fermentation, and in the case of *A. niger* until the sixth day of fermentation. They also concluded that SSF with filamentous fungi contributes to a greater recovery of lipids from apricot kernels and obtains oil with a high content of linoleic acid.

#### 3.3.2. Other Microorganisms

Besides filamentous fungi, yeasts and bacteria are also used in SSF. Filamentous fungi are more resistant to bacterial contamination, and yeasts and some types of bacteria have the ability to thrive when grown in low water activity environments. *Actinomycetes* such as *Streptomyces* sp. are resistant to extreme conditions and can colonize the substrate abundantly; thus, they are also used in SSF [98,99].

Bacteria are mostly used for enzyme production (various proteases, amylase, exo-polygalacturonase, mannanase, tannase, xylanase, lipase, etc.), and yeasts are known for the production of clorogenic acid, aromatics, etanol, etc. For example, *Bacillus* sp. BBXS-2 was used to produce protease and amylase in non-sterile open SSF. Wheat straw proved to be the best of several substrates used (sugarcane bagasse, wheat straw, rice straw, and rice husks). The activity of protease was about 12,200 U/g dry matter and amylase 6900 U/g dry matter after 5 days of fermentation. Produced enzymes were used as an additive to detergent, which increased its efficiency in removing starchy stains up to 2.5 times [100]. *Kluyveromyces marxianus* is a yeast that belongs to the GRAS (generally regarded as safe) group of microorganisms and is considered a microorganism that can grow on a variety of substrates and under extreme conditions. Therefore, it is frequently used in bioprocesses such as SSF [78,101]. Medeiros et al. [101] cultivated *K. marxianus* on five different substrates (apple pomace, cassava bagasse, sugarcane bagasse, sunflower seed bran, and giant palm bran) under SSF conditions to produce aroma compounds. Cassava bagasse and palm bran were found to be the best substrates for yeast growth, and the components produced in the highest amounts were ethyl acetate, ethanol, and acetaldehyde.

Data on the utilization of various AFIRs and their products after solid-state cultivation of bacteria (Table 4) and yeasts (Table 5) are reviewed in this paper.

## 4. Enzyme Production by SSF

During the biotransformation process of AFIRs for the purpose of producing various value-added products, biofuels, or animal feeds, the conversion of lignocellulosic biomass into fermentable sugars, sugar acids, and/or phenols is carried out by a complex enzymatic system of selected microorganisms [131].

SSF can offer significant benefits in the economic aspects of the enzyme production compared to SmF, since it uses low-cost and easily available substrates, such as lignocellulosic substrates, especially AFIRs [132]. Selection of substrate, microorganism, and process conditions has influence on desired enzyme(s) production. This section describes the catalytic activities of the most investigated enzymes produced by SSF by different microorganisms, and possible industrial application are given.

### 4.1. Lignocellulolytic Enzymes

Lignin is a complex, aromatic, and optically inert hydrophobic amorphous three-dimensional polymer consisting mainly of three different phenylpropane alcohols: *p*-coumaryl, coniferyl, and sinapyl. Their quantities depend on various factors, such as plant species, maturity, and the space localization in the cell [20]. Lignin is responsible for the structural rigidity of plants, their impermeability, and their resistance to microbial attacks and oxidative stress. Due to its properties, lignin is a major obstacle in the AFIR bioconversion process into valuable compounds [18]. The enzymatic system responsible for the fungal degradation of lignin is comprised of ligninases: phenol oxidases (laccase, EC 1.10.3.2) and peroxidases (manganese peroxidase (MnP), EC 1.11.1.13, lignin peroxidase (LiP), EC 1.11.1.7) [133].

Laccases are multi-copper glycoproteins that use molecular oxygen to oxidize various aromatic and non-aromatic compounds by a radical-catalyzed reaction mechanism. Laccase can be used in food and beverage industries for modification of color appearance, in the pulp and paper industry for delignification, and in the textile industry for textile bleaching or dye synthesis, as well as for many other purposes such as soil bioremediation, herbicide degradation, synthetic chemistry, cosmetics, and biosensors [134,135]. Laccases are found in higher plants, insects, prokaryotes, and fungi, but the most commonly used microorganisms in SSF for laccase production are white-rot fungi such as *T. versicolor*, *T. pubescens*, *Ganoderma lucidum*, and *Pleurotus eryngii*. Osma et al. [75] showed that banana peels can be a good substrate for the cultivation of *T. pubescens* under SSF conditions for laccase production. They indicated that by using this type of non-expensive substrates, it is possible to produce enzymes with higher activities at lower production costs. Produced laccase had a maximum activity of 1500 U/L and was found to be more efficient in decolorization of anthraquinone dyes compared to commercial laccase. Potato peel waste, pretreated with distilled water, is also one of the examples of economical substrates for the production of highly active laccase (6708.3 U/L) under SSF conditions with *P. ostreatus* [88].

Manganese peroxidase (MnP) belongs to the peroxidase family. It is an extracellular glycosylated heme enzyme that uses H_2_O_2_ to oxidize Mn^II^ to Mn^III^–chelate. This enzyme is mostly produced by numerous species of fungi (*Basidiomycetes*), especially white-rot fungi, and bacteria (*Actinomycetes*). It belongs to group of enzymes that have a significant role in efficient bioconversion of plant residues. MnP finds its use in various industries—paper, food, dye, textile, cosmetics, and many others [88].

Lignin peroxidase (LiP) is a water-soluble glycosylated enzyme that also uses H_2_O_2_ for catalysis. LiP is enzyme capable of producing radical cations through one-electron oxidation of nonphenolic aromatic compounds as well as phenolic aromatic compounds such as veratryl alcohol or 1,4-dimethoxybenzene [136]. This enzyme, like MnP, is produced mostly by filamentous fungi and participates in lignin degradation, having many applications in different industries [88,134]. The white-rot fungus *Inonotus obliquus* produces all three of the above ligninolytic enzymes (laccase, MnP, and Lip) under SSF conditions. Xu et al. [137] optimized process parameters such as pH, temperature, substrate moisture ratio, and inoculum level. Various lignocellulosic materials have been used as substrates (wheat bran, wheat straw, rice straw, peanut shell, sugarcane bagasse, cassava peel, birch branch, beech branch). Under optimal conditions, laccase, MnP, and LiP enzyme activities of 81.94 ± 7.55, 1603 ± 7.76, and 1500 ± 21.44 IU/g were obtained, respectively.

### 4.2. Cellulolytic Enzymes

Cellulose is an unbranched long polymer of β-D-glucose units linked by (1→4) glycosidic bonds to form cellobiose-repeating units in the cellulose chain. A numerous hydroxyl groups SSF can offer significant benefits on the inner and outer surface of cellulose-forming hydrogen bonds, while cellulose chains are interlinked by hydrogen bonds and Van der Waals forces. Owing to different orientations throughout the structure, cellulose molecules have different levels of crystallinity—low crystallinity (amorphous regions) and high crystallinity (crystalline regions) [20].

Cellulases are enzymes that have the ability to break cellulose and convert it into simple sugars. They include endoglucanases (1,4-β-D-glucan glucohydrolase), exoglucanases or cellobiohydrolases (1,4-β-D-glucan cellobiohydrolase), and β-glucosidases or cellobiases (β-D-glucoside glucohydrolase). Endoglucanases (EC 3.2.1.4) randomly hydrolyze internal glycosidic linkages (β-1,4 glucosidic bonds), resulting in shorter polymer chains and an increase of released number of reducing ends.

Endoglucanases find their application in the formulation of detergent compositions for increasing the production yield. They can also be used for improving the nutritive quality of products obtained in different food industry sectors (fruit processing industry; beer, oil, and bakery industries). They are known to be used in feed production [138] and in the textile and pharmaceutical industries as well [19].

Endoglucanases are mainly produced by fungi and bacteria cultivated on AFIRs. The most important producers of endoglucanases are given in Table 6. Exoglucanases (EC 3.2.1.91) or cellobiohydrolases (CBHs) catalyze cellulose hydrolysis to cellobiose units by acting on reducing and non-reducing end of the cellulose. Furthermore, released cellobiose units can be converted to glucose by β-glucosidase [139]. CBHs have tunnel-shaped active sites that accept only a substrate chain via its end terminal regions. It works by stinging the cellulose chain through the tunnel, removing the cellobiose units in a sequential manner [140]. The most important producers of exoglucanases are given in Table 6.

β-Glucosidases (EC 3.2.1.21) catalyze the hydrolysis of the β-glucosidic linkages, β-linked oligosaccharides, and oligosaccharides with the release of glucose. They reduce cellobiose-mediated repression and thus enable cellulolytic enzymes to be more effective [139]. Due to its ability of utilizing different glycosidic substrates, β-glucosidase is also an industrially important enzyme. It can be used for enzymatic hydrolysis of cellulose for different purposes: production of fermentable sugars, coproduction of functional foods, production of low-viscosity gellan foods, and improvement of food and beverage quality. The result of hydrolytic activity of β-glucosidase is the releasing of aglycone moiety, which has strong biological activity and can be used as antitumor agents in the prevention of coronary heart disease and cancer. Namely, the most of phenolic compounds from AFIRs exist in conjugated form with sugars linked to hydroxyl groups. This conjugation in the form of glucosides reduces their antioxidant potential since the availability of free hydroxyl groups on the phenol ring affects the resonance stabilization of free radicals. The reduced antioxidant activity has a direct impact on the weaker health functionality during the ingestion of these compounds in the body [141]. It has been recognized that bioaccessibility of high-molecular weight polyphenols (e.g., hydrolysable, condensed tannins), complex flavonoids conjugated with sugars and acetylated with hydroxycinnamic acids, are lower compared to aglycones (units without sugar) and low-molecular weight polyphenols [142]. Therefore, liberation of free phenolic compounds may improve their effect on the health functionality. The most important producers of β-glucosidases are given in Table 6. In addition to ligninolytic enzymes, the previously mentioned white-rot fungus *I. obliquus* also produces cellulolytic enzymes under SSF conditions. The maximum activities of the enzymes carboxymethylcellulase, filter paper cellulase, and β-glucosidase obtained under optimal process conditions using wheat bran as substrate at 40% inoculum, pH 6.0, and substrate/moisture ratio of 1:2.5 were 27.15, 3.16, and 2.53 IU/g, respectively [137].

*Trichoderma* is one of the microorganisms that have been extensively studied for the production of various industrially important enzymes, mainly cellulase, exoglucanase, and β-glucosidase under SSF conditions using different AFIRs as substrates. The studies conducted by Shazhadi et al. [143] aimed at hyperproduction of exoglucanase and β-glucosidase using a low-cost and readily available corn stover substrate. Optimization of process conditions (substrate amount 15 g; 50% *w*/*w* moisture, 6 mL inoculum, pH 6.0, 35 °C) for successful growth of co-culture of *T. viride* and *G. lucidum* on corn stover resulted in production of exoglucanase and β-glucosidase enzymes and their activities of 485 ± 6.5 U/mL and 255 ± 3.3 U/mL after 5 days of incubation, respectively. They also investigated the influence of additional carbon and nitrogen sources regulating enzyme synthesis during growth of white-rot fungi, and the combination of glucose and ammonium sulfate proved to be the best in the production of exoglucanase and β-glucosidase.

### 4.3. Hemicellulolytic Enzymes

Hemicellulose is a complex of polysaccharide matrixes composed of different units of sugars (xylans, glucans, xyloglucans, callose, mannans, and glucomannans). It is the second most abundant polysaccharide in plant cell wall. Xylan is the most abundant hemicellulose polymer, constituting around 70% of hemicelluloses. Galcto(gluco)mannans and xyloglucans are another two major hemicelluloses in plant cell wall. In order to degrade such a complex material, microorganisms should have ability to produce a large set of hemicellulases, which act in interaction.

Hemicellulases include xylanases (EC 3.2.1.8), β-mannanases (EC 3.2.1.78), arabinofuranosidases (EC 3.2.1.55), and β-xylosidases (EC 3.2.1.37). Endo-1,4-β-xylanases (also called xylanases, endoxylanases, 1,4-D-xylan-xylanohydrolases, endo-1,4-β-D-xylanases, β-1,4-xylanases, and β-xylanases) belong to the glycosil hydrolase family. They catalyze the hydrolysis of 1,4-glycosidic linkages between xylose residues in the backbone of xylans [161]. Since xylan is the major part of hemicellulose, xylanase is the key enzyme for depolymerization of hemicellulose components [72,162]. For complete hydrolysis of xylan to be achieved, the following enzymes are required: α-arabinofuranosidase, α-glucuronidase, acetylxylan esterase, and hydroxycinnamic acid esterase split side residues from the xylan backbone. Xylanases find their application in the food industry (brewing, wine production, juice clarification, baking), textile industry, and bioremediation [157,158,161]. Additionally, they can be applied in the pulp and paper industry, which results in a reduced amount of chlorine and chlorine dioxide commonly used for bleaching paper pulp. The most important producers of xylanases are given in Table 6.

Tomato pomace is a waste available in large quantities, and its chemical composition contains proteins, lipids, carbohydrates, amino acids, carotenoids, and minerals. Umsza-Guez et al. [159] used this waste as a substrate in SSF for xylanase production. Fermentation was carried out in a conical flask and a laboratory scale plate-type SSF reactor by *A. awamori*. In conical flasks, the maximum activity of xylanase was reached between the fourth and eighth day of fermentation (about 100 IU/g_ds_), while in the plate-type SSF reactor, the maximum activity was reached on the fifth day of fermentation (195.92 ± 11.0 IU/g_ds_).

Some studies have shown that co-cultivation of compatible microorganisms can enhance enzyme biosynthesis. Gupta et al. [103] studied the co-cultivation of SSF bacteria (*Bacillus* sp. and *B. halodurans* FNP135) producing xylanase and laccase. They used wheat bran as substrate. Under optimized conditions (pH 10.5, inoculum size 10+10%, moisture/substrate ratio 0.8:1), a significant increase in the production of xylanase (1685 IU/g) and laccase (2270 IU/g) was obtained. The mixed enzyme preparation was found to be effective in bio-bleaching of craft pulp.

Some researchers are concerned with the purification and characterization of the enzymes produced, as this is an important step that provides insight into enzyme properties and helps determine potential applications. David et al. [104] optimized the production of mannanase and protease using *Bacillus nealsonii* under SSF conditions on wheat bran as substrate. The protease was purified by standard protein purification procedures that include methods such as ammonium sulfate precipitation, gel filtration chromatography, and ion exchange chromatography. Each step was performed at 4 °C, and enzyme volume, protease activity, and protein content were determined after each step. The combination of mannanase and protease from *B. nealsonii* was found to be effective in removing various stains when used as detergent additive.

## 5. Production of Phenolic Compounds and Other Value-Added Compounds

AFIRs are rich in nutrients and bioactive compounds. Therefore, these residues have potential applications in SSF processes for the obtainment of beneficial compounds such as phenolic compounds, organic acids, flavor, and aroma compounds (Table 3, Table 4 and Table 5), which possess antioxidative, anti-inflammatory, antiallergic, antiviral, anticancer, antimicrobial, and antimutagenic properties [2]. A trend is to enrich food products with AFIRs, primarily because of the high content of dietary fibers and bioactive polyphenolic compounds, which increase the nutritional value and help in diseases prevention, but also positively affect stability, organoleptic properties, and technological properties of the final product [163].

Phenolic compounds represent the important group of bioactive compounds from plant material. They are the most abundant antioxidants in the human diet. Their structure consists of an aromatic ring, containing one or more hydroxyl substituents. The number and position of the hydroxyl groups, and the nature of substituents on the aromatic rings, affect the physiological properties of phenolic compounds. These compounds show a broad spectrum of physiological properties, such as anti-allergenic, anti-artherogenic, anti-inflammatory, anti-microbial, antioxidant, anti-thrombotic, cardioprotective, and vasodilatory effects [164]. Generally, they can be divided into three main groups, namely, phenolic acids (hydroxycinnamic acids, hydroxybenzoic acids), flavonoids (flavones, flavonols, flavanols, anthocyanins), and tannins (hydrolysable and nonhydrolyzable or condensed tannins) [165]. AFIRs are a cheap and rich source of potentially functional ingredients, such as phenolic compounds, thus promoting a circular economy concept. For example, after the processing of apples, it is estimated that 82–99% of the original polyphenols remain in apple pomace [166].

Lignin fraction of AFIRs contains various phenolic compounds, mainly phenolic acids such as ferulic, *p*-coumaric, syringic, vanillic, and *p*-hydroxybenzoic [39]. Simple phenolic compounds from biological materials can usually be isolated by extraction with organic solvents, while non-extractable highly polymerized proanthocyanidins and phenol complexes with proteins, fibers, and polysaccharides have to be hydrolyzed or degraded beforehand. The methods used for this are acid hydrolysis, which is environmentally unacceptable, and enzymatic hydrolysis, which is economically inconvenient [82]. On the other hand, phenolic compounds can be recovered by SSF, during which the microorganisms synthesize enzymes involved in breakdown of complex lignocellulosic material and release of these valuable compounds [11] (Table 7).

Ajila et al. [86] studied the ability of *P. chrysosporium* to release phenolic antioxidants from apple pomace using SSF and examined the effectiveness of various extraction parameters on antioxidant extraction (Table 7). They concluded that the SSF improved not only the nutraceutical properties of apple pomace but also the antioxidant activity wherein the increase in these values is dependent on the extraction conditions. The IC_50_ values obtained from the polyphenol extract by optimum extraction conditions (microwave-assisted extraction at 40 °C for 30 min with 80% acetone) was 20.12 μg_db_ of sample for apple pomace and 12.24 μg_db_ of sample for fermented apple pomace. Bucić-Kojić et al. [82] investigated the recovery of phenolic acid and enzyme production from biologically treated corn silage by white-rot fungus *T. versicolor*. According to obtained results, increments in extraction yield of caffeic acid, vanillic acid, *p*-hydroxybenzoic acid, and syringic acid were observed in fermented grape pomace (Table 7). Further, Martínez-Ávila et al. [167] reported that the cultivation of *Aspergillus* and *Penicillium* strains on grape waste in SSF can improve the production of phenolic antioxidant compound (gallic acid). Pomegranate peel and creosote bush leaves were successfully used as a substrate for ellagic and gallic acid production by *A. niger* GH1 [168]. In the recent period, large-scale chokeberry cultivation has increased due to its high polyphenolic content and antioxidant activity. SSF of the chokeberry pomace by *A. niger* and *R. oligosporus* and its influence on the content of phenolic compounds, antioxidant activity, and lipid composition were investigated by Dulf et al. [57]. They concluded that SSF leads to an increase of total phenolic and total flavonoid contents, as well as a formation of lipids with better nutritional quality characteristics. On the same substrate, SSF with *Lentinus edodes* resulted in an increase in the ellagic acid content [141].

Organic acids are used as preservatives in food and beverage production since they can prevent spoilage and prolong the shelf life of food. Sharma et al. [93] have shown feasibility of SSF gluconic acid production from sugarcane molasses by *A. niger* ARNU-4 using tea waste as substrate.

In the research conducted by Nimnoi and Lumyong [123], fungus *M. purpureus* was cultivated on corn meal, peanut meal, and coconut residue and soybean meal for the purpose of red pigment production.

Fungi and bacteria have been reported as microorganisms with great ability to produce flavor and aroma compounds during SSF. Production of fruity flavors by fungi *Ceratocystis fimbriata* under SSF conditions, using a coffee husk as a substrate, was tested by Soares et al. [94]. *Kluyveromyces marxianus* proved to be suitable for producing value-added aroma compounds by SSF using a sugarcane bagasse and sugar beet molasses [78]. Application of SSF for the production of biosurfactants, biolubricant oleogels, and biodegradable polymers (PHAs) has been shown to be effective using certain species of bacteria [110,112,118].

## 6. Biofuel Production

Due to the increasing trend towards the production of biofuels as a substitute for fossil fuels, environmentally friendly methods for their production are being developed. The first-generation biofuels are usually produced from crops (sugar cane, sugar beet, wheat, rice, sunflower oil, etc.), but from a sustainability point of view, the main limitation of production of first-generation biofuels is food and energy competition [169]. Therefore, there is a large amount of interest in the development and use of second- and third-generation biofuels in agriculture, forestry, and industrial production, as well as in advanced sustainable waste management. The second-generation biofuels are mainly directed towards the production of biofuels from reusable materials such as municipal solid waste, agro-industrial waste, and sewage sludge, which can actually produce biofuels such as bioethanol, biodiesel, bioalcohols, biogas, and biohydrogen [9,169].

Complex chemical structure of biomass (e.g., AFIRs) requires the pretreatment of such lignocellulosic material, which is a pivotal step before being involved in the hydrolysis and fermentation process. Delignification and detoxification are two main targets of ligninolytic enzymes in biofuel production. Delignification presents reduction of lignin content in biomass, while detoxification means reduction of toxic components present in biomass hydrolysate [9]. Tišma et al. [55] investigated the effect of pretreatment of corn silage in biogas productivity. Corn silage pretreated with white-rot fungus *T. versicolor* increase biogas productivity. Jain et al. [68] found that bioprocessing of switchgrass under SSF conditions by *C. phytofermentans* may represent an alternative to SmF in the production of reducing sugars that are metabolized to ethanol and acetate. *Clostridium phytofermentans* is considered a suitable anaerobic mesophilic bacterium for biological treatment of lignocellulosic biomass because it produces numerous enzymes that participate in degradation of complex carbohydrates to simple sugars.

## 7. Feed Production

The quality of feed is an imperative of modern livestock agriculture. There is a great increase of need for feed as a consequence of world population growth and the need for food of animal origin.

Therefore, many researchers focus on manipulation with unconventional feed sources, such as lignocellulosic biomass with microorganisms. AFIRs remaining after cereal (e.g., corn, barley, wheat, and rye) processing possess binding properties because of starch they contain. However, due to the low nitrogen and mineral content, as well as the reduced digestibility associated with high lignin, cellulose, and hemicellulose content, direct utilization of AFIRs for ruminant feeding is limited [170]. To improve the access of microbial hydrolytic enzymes to cellulose and hemicellulose for digestibility enhancement, one must break lignin carbohydrate linkages in the plant cell wall. SSF could bring benefits in terms of enhancement of digestibility of those types of materials, but this bioprocess has not yet fully developed and much effort is still needed for production animal feed by SSF on a large scale. White-rot fungi such as *Phanerochaete chrysosporium*, *Pleurotus* sp., *L. edodes*, *Coriolus versicolor*, *Phlebia* sp., *Ceriporiopsis subvermispora*, and *Ganoderma* sp. have been applied for SSF of various AFIRs (wheat straw, olive mill solid waste, madake bamboo, tanniniferous lespedeza plants, oil palm fronds etc.) to produce animal feed of better quality through improved digestibility, enhanced palatability, and availability of fermentable energy to ruminal microbes [170]. The cultivation of different microorganisms on AFIRs has great potential in the production of biomass suitable for use as animal feed supplement, since such dry biomass may contain 30–60% protein, up to 40% carbohydrate, and 20–50% oil [171]. The composition of dry biomass depends on cultivation method (submerged or solid-state fermentation), growing conditions, choice of microorganism, and type of organic substrate [171]. In addition to microbial proteins or single-cell proteins, various enzymes that can be used as a supplement in animal feed can be manufactured by SSF. Enzymes such as xylanases, pectinases, and amylases are used in animal feed primarily to increase nutrient digestibility. Cellulases, which stimulate fiber degradation, and tannins, which hydrolyze tannins, are also used. Studies have shown that in ruminants, lower concentrations of tannins in the diet result in increased nitrogen assimilation, which in turn leads to higher growth rates and increased milk production. The enzyme phytase is also important for improving the nutritional value of animal feed, as its mechanism releases phosphorus, which is an important nutrient for proper animal development [172].

The problems in modern livestock agriculture are that animal feed and feed materials can be contaminated with undesirable substances (e.g., heavy metals, mycotoxins), which may originate from the environment and/or the production process. When production animals consume such contaminated feed, the contaminants may transfer to food of animal origin, such as liver, meat, and milk. In the European Union (and most of the rest of the world), legislation is in place to manage the feed and feed material contamination. Official limits and guidelines indicate safe levels of each contaminant, often per feed (material) group. Moreover, procedures for control of the presence of the contaminant are prescribed, e.g., the number of samples to be taken for which contaminants. Within the European Union (EU), Regulation (EC) No. 882/2004 provides rules for the official controls that have to be performed to ensure the verification of compliance with feed and food law, animal health, and animal welfare rules [173].

The possible solution to remove mycotoxins is the use of selected white-rot fungi, such as, e.g., *T. versicolor* culture filtrate, which was shown to be a very promising tool for aflatoxin B1 reduction of contaminated maize in animal feed. Studies on the use of *T. versicolor* in the animal nutrition indicate its highly relevance in the term of sustainable feed production [69].

## 8. Conclusions and Future Prospects

AFIRs are rich natural sources of various nutrients and bioactive compounds. Although these residues can cause serious economic and environmental problems due to unacceptable disposal, large quantities of residues are considered as potential for reuse and as energy sources. Their availability and low cost could lead to more economical industrial scale processes. SSF is an environmentally friendly bioprocess that does not use expensive AFIRs as substrates and can offer significant advantages in economic terms for the production of valuable compounds (enzymes, polyphenolic compounds, biofuels, enriched animal feed, etc.). It is also an alternative to the pretreatment process for the low-cost production of sustainable biofuels, which poses a challenge for the commercialization of the production process. The main drawback of this technology is that the hydrolysis of lignocellulosic components in some feedstocks is too slow, which prevents this method as a potential pretreatment method at industrial level. To speed up the process and make it more efficient, it is possible to use a combination of another pretreatment method (physical, chemical) with the biological pretreatment. However, the development and establishment of combined methods still requires a large amount of research and work to reach full efficiency. Numerous studies have led to great progress in the development of SSF processes from laboratory scale to scale-up and bioreactor application. With the increasing competition in the industry, optimization of the process and bioreactor design are important factors in the field of future research.

## Figures and Tables

**Table 1:** Chemical composition of common agricultural residues.

Agricultural Residues	Lignin, %_db_	Cellulose, %_db_	Hemicellulose, %_db_	Ash, %_db_	Reference
Barley husk	22.0	39.0	12.0	7.0	[22,23]
Barley straw	9.6–13.8	33.8–46.8	21.9–30.0	4.4	[5,24,25]
Corn cob	6.1	33.7	31.9	8.5	[5,24]
Corn stalks	7.0–7.3	35.0–39.0	16.8–42.0	24.9	[5,25]
Oat straw	4.1–23.6	31.7–39.4	23.3–28.2	3.2	[5,24,26]
Rice straw	8.3–9.9	19.6–36.2	19.0–50.4	14.7	[5,25,27]
Rye straw	19.0–30.8	37.4–37.6	30.5	5.7	[5,26,28]
Soybean stalks	19.8	34.5	24.8	ND	[29]
Spelt straw	14.8	38.3	24.3	5.7	[30]
Sunflower seed hulls	29.4	29.4	29.4	2.1	[29,26]
Sunflower stalks	13.4–17.5	38.5–42.1	29.7–33.5	8.6–9.2	[5,27]
Wheat straw	8.9–22.1	32.9–49.8	23.7–25.0	3.6–4.7	[5,24,25,31,32]

%_db_—percentage of the dry solids (dry basis); ND—not determined.

**Table 2:** Chemical composition of common food industry residues.

Food Industry Residues	Lignin, %_db_	Cellulose, %_db_	Hemicellulose, %_db_	Protein, %_db_	Ash, %_db_	Reference
Apple pomace	14.8–22.4	47.5	27.8	6.0–7.0	1.1–5.1	[37,38]
Brewers spent grain	4–27.8	13.14–16.8	28.4–39.0	23.4–27.4	3–5	[39,40]
Flax oil cake	6.0	8.2	4.6	32.8	5.3	[41]
Grape pomace	11.6–41.3	9.2–14.5	4.0–10.3	7.0–23.5	4.7–9.5	[42,43]
Hemp oil cake	16.7	22.5	14.2	24.8	7.5	[41]
Hull-less pumpkin oil cake	0.7	4.4	6.7	38.3	7.8	[41]
Olive mill waste	13.3–15.8	24.8–33.8	13–16.3	6.7–7.2	2.5–8.9	[44,45]
Rice bran	24.8	34.0	28.2	5.8–8.3	ND	[46]
Rye bran	3.5–4.4	5.0–6.0	ND	14.4–18	2.8–6.2	[22,47]
Sugarcane bagasse	18.9–26.1	36.9–45.7	25.60–29.58	2.18	2.84	[48,49]
Wheat bran	3.0–5.0	9.0–12.0	38.9	9.6–18.7	0.04–8.1	[50,51,52]

%_db_—percentage of the dry solids (dry basis); ND—not determined.

**Table 3:** Filamentous fungi used for the production of enzymes and other value-added products during SSF on different food industry waste or by-products.

Division	Microorganisms	Substrates	Products	Reference
*Basidiomycota*	*Trametes versicolor*	tomato pomace	laccase, xylanase, protease	[84]
	*Trametes versicolor*	brewer spent grain	laccase, polyphenols	[55]
	*Trametes versicolor*	corn silage	laccase, manganese peroxidase, caffeic acid, vanillic acid, *p*-hydroxybenzoic acid, syringic acid	[82]
	*Trametes versicolor*	barley husk and egg shell	laccase	[85]
	*Trametes pubescens*	banana skin	laccase	[75]
	*Trametes hirsuta*	grape seeds	laccase	[59]
	*Phanerochaete* *chrysosporium*	apple pomace	phenolic antioxidants	[86]
	*Pleurotus ostreatus*	potato peel, wheat bran, tomato pomace, fresh pineapple residue, rice straw	ligninolytic enzymes, xylanase, protease, bioactive phenolic, antioxidant compound	[84,87,88,89]
	*Pleurotus ostreatus*	apple bagasse, agave mezcalero bagasse	phenolic compounds, flavonoids, triterpenes	[90]
	*Bjerkandera adusta*	wheat bran	carboxymethil cellulase, manganese peroxidase, laccase, xylanase	[87]
*Ascomycota*	*Aspergillus niger*	plum fruit by-products	higher lipid recovery, isoquercitrin	[76]
	*Aspergillus niger*	apricot pomace	neochlorogenic and chlorogenic acids, rutin, quercetine-3(6“acetyl-glucoside)	[83]
	*Aspergillus niger*	granadilla seeds flour, moringa leaves	phenolic compounds	[91,92]
	*Aspergillus niger*	sugar molasses	gluconic acid	[93]
	*Aspergillus niger* *Aspergillus ibericus*	olive pomace, winery waste	bioactive compounds	[11]
	*Aspergillus niger* *Rhizopus oligosporus*	chokeberry pomace	cinnamic acid, flavonols	[57]
	*Ceratocystis fimbriata*	coffee husk	fruit flavor	[94]
	*Thermoascus* *aurantiacus*	orange, sugarcane bagasse, wheat bran	pectinases	[95]
	*Thermomyces* *lanuginosus*	hull-less pumpkin oil pomace	lipase	[96]
*Zygomycota*	*Rhizophus oligosporus*	plum fruit by-products	higher lipid recovery, isoquercitrin	[76]
	*Rhizophus oligosporus*	apricot pomace	neochlorogenic and chlorogenic acids, rutin, quercetine-3(6“acetyl-glucoside)	[83]
	*Actinomucor elegans* *Umbelopsis isabellina*	grape pomace	*γ*-linolenic acid and carotenoids	[12]
	*Rhizopus delemar* F2	apple pomace	carbohydrase production	[97]
	*Mortierella alpina*	oilseed cakes	increased nutritional value of oilseed cakes	[56]

**Table 4:** Bacteria used in SSF for production of different compounds.

Microorganisms	Substrates	Products	Reference
*Actinobacillus succinogenes*	fruit and vegetable hydrolysate	succinic acid	[102]
*Bacillus halodurans* FNP 135	wheat bran	xylanase, laccase	[103]
*Bacillus nealsoni* PN-11	wheat bran	mannanase, protease	[104]
*Bacillus subtilis* BBXS-2	sugarcane bagasse, wheat straw, rice straw, rice husk	protease, amylase	[100]
*Bacillus subtilis* DM-04	potato peels, mustered oil cake, wheat bran, rice bran, banana leaves, tea leaves	alkaline protease	[105]
*Bacillus subtilis* RCK	wheat bran	exo-polygalacturonase	[106]
*Bacillus thuringiensis*	municipal solid waste mixed with wood chips	compost with enhanced biopesticide properties	[107]
*Brevibacterium casei* MSA19 *Serratia rubidaea* SNAU02 *Nocardiopsis lucentensis* MSA04	oil seed cake, wheat bran, tannery treated sludge, tannery pretreated sludge, treated molasses and pretreated molasses, groundnut oil cake, coconut oil cake, gingelly oil cake, castor oilcake, palm oil cake, sunflower oil cake and mahua oil cake	biosurfactants	[108,109,110]
*Clostridium phytofermentans*	switchgrass	reducing sugars that are further metabolized to ethanol and acetate	[68]
*Cupriavidus necator*	soy cake, babassu cake	biodegradable polymers (polyhydroxyalkanoates, PHAs)	[111,112]
*Enterococcus faecalis* M2	wheat bran	improved antioxidant properties and nutritional quality of wheat bran	[58]
*Lactobacillus amylophillus* GV6	wheat bran	L-(+)-lactic acid	[113]
*Lactobacillus casei* *Lactobacillus fermentum*	broken dried chestnuts	improved nutritional composition	[114]
*Lactobacillus* sp. ASR-S1	tamarind seed powder, wheat bran, palm kernel cake, coffee husk	tannase	[115]
*Pseudomonas* sp. BUP6	deoiled cake of groundnut, gingelly, coconut, soybean and cotton seed	lipase	[116]
*Streptococcus thermophiles* *Lactobacillus bulgaricus*	wheat bran	improved nutritional, physical and flavor properties of wheat bran	[77]
*Streptomyces* sp.	cassava residues, rapeseed cake, mushroom residues, bean cake, wheat bran, rice bran, wheat straw	biolubricant oleogels, ε-poly-lysine (food preservative)	[117,118]
*Streptomyces* sp.	soybean meal ground, wheat bran	L-asparaginase	[119]
*Streptomyces* sp. MDG147	wheat straw	biolubricant oleogels	[118]

**Table 5:** Yeasts used in SSF for production of different compounds.

Microorganisms	Substrates	Products	Reference
Active dry yeast (commercial baker’s yeast with high sugar tolerance)	wheat bran	improve the nutritional, physical and flavor properties of wheat bran	[77]
*Kluyveromyces marxianus* ATCC 10022 *Pichia kudriavzevii*	sugarcane bagasse	2-phenylethanol, 2-phenethyl acetate	[120,121]
*Kluyveromyces marxianus*	sugarcane bagasse, sugar beet molasses, cassava bagasse, giant palm bran	aroma compounds	[78,101]
*Kluyveromyces marxianus* NRRLY-7571	sugarcane bagasse, corn steep liquor, soybean meal, sugarcane molasses	inulinase	[122]
*Monascus purpureus*	corn meal, peanut meal, coconut residue and soybean meal	red pigment	[123]
*Meyerozyma guilliermondii* *Candida glaebosa* *Cryptococcus victoriae* *Leucosporidium scotti*	inert support of polyurethane and addition of nutrient medium	L-asparaginase, protease	[124]
*Pichia pastoris* *Kluyveromyces marxianus* *Kluyveromyces lactis* *Saccharomyces cerevisiae* *Candida* sp. *Aureobasidium pulluans* *Cryptococcus aureus* *Schwanniomyces castellii* *Endomicopsis burtonii*	polyurethane foam, apple pomace, cassava bagasse, sugarcane bagasse, sunflower seeds, giant palm, corn grits, wheat bran, soy bran, soy peel, corn cob	proteins and secondary metabolites	[81]
*Saccharomyces cerevisiae*	coffee pulp	chlorogenic acid	[125]
*Saccharomyces cerevisiae*	corn cob residues	ethanol	[126]
*Saccharomyces cerevisiae* PM-16	grape pomace, fresh fruit and vegetable residues, corn cob residues	ethanol	[126,127,128]
*Saccharomyces cerevisiae* *Schwanniomyces occidentalis* *Scheffersomyces stipitis*	fresh fruit and vegetable residues	ethanol	[127]
*Yarrowia lipolytica*	luffa sponge, okara, dried loofah sponge, wheat bran, corncob, buckwheat husk, sugarcane bagasse	γ-decalactones, erythritol	[79,129]
*Zygosaccharomyces rouxii*	oatmeal and wheat bran	glutaminase	[130]

**Table 6:** Valorization of different AFIRs for the production of enzymes from different microorganisms.

Enzymes		Microorganism	Substrate	Reference
Lignolytic	laccase	*Trametes versicolor*	corn silage, brewers’ spent grain, barley husk	[82,85,144]
		*Trametes pubescens*	banana skin	[75]
		*Pleurotus eryngii*	peach waste	[145]
		*Aspergillus flavus* PUF5	dried ridge gourd peel	[146]
		*Ganoderma lucidum*	wheat bran	[147]
		*Lysinibacillus* sp.	wheat bran	[148]
	manganese peroxidase lignin peroxidase	*Inonotus obliquus*	birch branch, beech branch, rice straw, wheat straw, wheat bran, sugarcane bagasse, cassava peel, peanut shell	[137]
Cellulolytic	cellulase endoglucanase exoglucanase	*Trichoderma* sp.	corn cob, wheat bran	[149]
		*Penicillium roqueforti*	rice husk	[150]
		*Aspergilus fumigatus*	wheat straw	[151]
		*Thermoascus aurantiacus*	Jatropha deoiled seed cake	[138]
		*Aspergillus fumigatus*	wheat straw	[152]
		*Trichoderma viride* *Ganoderma lucidum*	corn stover	[143]
	cellobiase	*Humicola insolens*	paddy straw, soybean pod husk, sugarcane bagasse, groundnut shells, corn stalks and pigeonpea pod husk	[153]
	*β*-glucosidase	*Lichtheimia ramosa*	wheat bran, soy bran, corn cob, corn straw, rice peel, sugar cane bagasse	[154]
		*Thermoascus aurantiacus* *Aureobasidium pullulans*	wheat bran, soy bran, soy peel, corn cob, corn straw	[155]
		*Trichoderma viride* *Ganoderma lucidum*	corn stover	[143]
Hemicellulolytic	xylanase	*Aspergillus oryzae*	wheat bran	[72]
		*Aspergillus tubingensis*	wheat straw, sorghum straw	[156]
		*Bacillus stearothermophilus*	wheat bran	[157]
		*Aspergillus niger*	rice straw	[158]
		*Aspergillus awamori*	tomato pomace	[159]
		*Thermomyces lanuginosus*	wheat bran	[160]
		*Humicola insolens*	paddy straw, soybean pod husk, sugarcane bagasse, groundnut shells, corn stalks and pigeonpea pod husk	[153]

**Table 7:** Production of phenolic compounds from some food industry residues by SSF.

Products	Conditions	Remarks	Reference
Total polyphenolic compounds from apple pomace	Substrate: apple pomace, treated with inducers: copper sulphate (2 mM), veratryl alcohol (2 mM) and Tween-80 (0.1%); pH 4.5; autoclaved (121 °C, 30 min), moisture content 72% *w*/*v*. Microorganism: *P. chrysosporium*, inoculation with spore suspension (2.5 × 10^6^ spores/g of solid). SSF: carried out in flasks, in controlled environment at 37 ± 1 °C for 14 days. Extraction (optimization): - type: UAE (in ultrasonication bath,) or MAE (in sealed green chem Teflon reactor vessel, pressure of 692 kPa, power 400 W). - solvent: water, or 60%, 70%, or 80% ethanol; acetone; or methanol. - temperature: 30, 40, 50, 60, 70, or 80 °C. - interval: 20, 30, or 40 min (UAE); 5, 10, or 15 min (MAE). - effect of surfactant: different concentrations of Tween-20 (0.1%, 1%, 2%, and 5% in *v*/*v* with water). After the extraction, sample mixture was centrifuged at 9268× *g* for 20 min to obtain the supernatant for further determination of total phenolic content (at 725 nm) and free radical scavenging activity (DPPH method at 517 nm).	The phenol content was higher in the fermented apple pomace, and the antioxidant activity correlated with the increase in polyphenol content, with both values depending on the type of solvent, extraction temperature, extraction time, and method used.	[86]
Individual polyphenolic compound from grape pomace	Substrate: corn silage, particle size 1.0–2.0 cm; autoclaved (121 °C, 20 min). Microorganism: *T. versicolor* TV-6, cultivated on PDA medium for 7 days at 27 °C; five mycelial plugs (diameter 1 cm) suspended in 10 cm^3^ of sterile water (inoculum). SSF: performed in laboratory jars at 27 °C for 5, 9, 13, and 20 days. Extraction: milled dry substrate after SSF was extracted by 50% ethanol with solid/liquid ratio 1:40, in a shaking-water bath at 80 °C by (200 rpm) for 120 min. After the extraction, samples were centrifuged for 10 min at 10,000× *g* in order to obtain liquid extracts for further UHPLC analysis of phenolic acids.	After 20 days of corn silage treatment with *T. versicolor*, 10.4-, 3.4-, 3.0-, and 1.8-fold increments in extraction yield of syringic acid, vanillic acid, *p*-hydroxybenzoic acid, and caffeic acid, respectively, were reached.	[82]
Phenolic antioxidants from grape waste	Substrate: grape waste, dehydrated at 60 °C/24 h, pulverized (30-mesh), stored at 22 °C. Microorganism: different fungal strains: *A. niger* GH1, PSH, Aa-20, ESH; *Penicillium pinophilum* ESH2, ESH3; *Penicillium purpurogenum* GH2; inoculation with 2 × 10^7^ fungal spores per gram of solid support. SSF: performed in tray reactor at 30 °C/60 h. Assay: total antioxidant activity of the extracts was tested by two different free radical (DPPH· and ABTS·+) inhibitions; free gallic acid content was estimated by HPLC.	The extracts of grape waste enhanced their free radical scavenging and preserved the capacity to avoid the lipid peroxidation after SSF. Gallic acid is not the only phenolic compound related to the free radical scavenging and antioxidant properties of the fermented samples.	[167]
Phenolic antioxidants from pomegranate peels	Substrate: pomegranate peels, cleaned, dried at 60 °C/48 h, pulverized, stored at room temperature in black bags. Microorganism: *A. niger* GH1; inoculation with 2 × 10^7^ spores/g of plant material, or substrate impregnated with culture broth. SSF: carried out in flasks at 30 °C for 96 h. Assay: tannins were analyzed using a spectrophometric method; concentration of gallic and ellagic acids was determined by HPLC.	The ellagic acid was accumulated considerably in pomegranate peels after fungal fermentation, which demonstrated that the high level of hydrolysable tannins in pomegrante peel tannins are mainly ellagitannins.	[168]
Phenolic antioxidants from chokeberry pomace	Substrate: chokeberry (cultivar “Nero”) pomace, dried < 40 °C, ground (0.5–1 mm), stored at 18 °C; moisturized (65%) with a nutrient solution (containing yeast extract and glucose), pH 5.5; autoclaved at 121 °C/30 min. Microorganism: *A. niger* ATCC-6275 and *R. oligosporus* ATCC-22959; inoculating cultures were produced by growing the strains on fresh PDA at 27 °C for 10 days, and spore inoculum was prepared by washing the agar surface with sterile distilled water. SSF: was carried out in in Erlenmeyer flasks at 30 °C for 12 days; substrate was inoculated with spore suspension 2 × 10^7^ spores/g of solid. Extraction: in an ultrasonic bath for 30 min at 40 °C with solvent mixture (hydrochloric acid: methanol: water in the ratio 1: 80: 19). The mixtures were centrifuged (4000× *g* for 10 min); supernatants were filtered and evaporated under vacuum and then stored in methanol (4 °C) until analysis (total phenolics, flavonoids, and anthocyanins; individual phenolics; antioxidant activities).	The extractable phenolics increased more than 1.7-fold during both fermentation processes, and a similar trend was observed for total flavonoids. The free radical scavenging ability of phenolic extracts were significantly enhanced during the SSFs. The amounts of flavonols and cinnamic acids increased while the concentrations of glycosylated anthocyanins decreased substantially.	[57]
Water-soluble phenolic antioxidants from cranberry pomace	Substrate: freshly pressed cranberry pomace, vacuum-dried and stored in a refrigerator. Microorganism: *Lentinus edodes* was maintained on PDA slants and Petri plates at 4 °C and sub-cultured. The fungus was resuscitated by transferring onto a PDA plate and cultured at room temperature 20 days before use. SSF: carried out in in Erlenmeyer flasks at 28 °C for 25 days (cranberry pomace + calcium carbonate + water + ammonium nitrate or fish protein hydrolysate was autoclaved at 121 °C for 20 min and the vegetative mycelia from one PDA plate were inoculated into flasks). Extraction: distilled water or 95% ethanol was added to fungus–pomace flask and the culture was homogenized for 1 min and then centrifuged at 15,000× *g* at 4 °C for 20 min and then filtered.	There was an increase in the extractable phenolic content. Both phenolics and antioxidant capacity correlated with the increase in the β-glucosidase activity, showing that the enzyme may play an important role in the release of phenolic aglycones from cranberry pomace and, therefore, increase the antioxidant capacity.	[141]

UAE—ultrasonic-assisted extraction; MAE—microwave-assisted extraction; PDA—potato dextrose agar; DPPH—1,1-diphenyl-2-picrylhydrazyl radical; ABTS·+—2,2′-azino-bis(3-ethylbenzothiazoline-6-sulfonic acid) radical; UHPLC—ultra-high-performance liquid chromatography; HPLC—high-performance liquid chromatography.

## Data Availability

The data presented in this study are available on request from the corresponding author.
